# PLWH treated with modern ART and high CD4 T cell counts: no evidence of HIV-associated vasculopathy measured by extra- and intracranial ultrasound

**DOI:** 10.1007/s13760-023-02339-2

**Published:** 2023-07-25

**Authors:** Mathias Fousse, Matthias Heit, Klaus Fassbender, Dominic Kaddu-Mulindwa

**Affiliations:** 1 Department of Neurology, Saarland University Medical School, Kirrberger Str. 100, 66421 Homburg, Germany; 2 Department of Hematology and Oncology, Clinical Immunology, Rheumatology, Saarland University Medical School, Kirrberger Str. 100, 66421 Homburg, Germany

**Keywords:** Ultrasound, HIV-associated cerebral vasculopathy, Cerebral reserve capacity (CRC), Breath Holding-Index (BHI), Intima–media thickness (IMT), Antiretroviral therapy (ART)

Increased prevalence of atherosclerotic cardiovascular diseases in people living with HIV (PLWH) might not be the only underlying pathophysiologic cause for the increased rate of ischemic stroke [1, 2].

Almost 20 years ago, impaired cerebrovascular reserve capacity without the presence of manifest vascular stenoses was considered to indicate a causative cerebral vasculopathy [1, 2].

Recently, we compared PLWH to healthy controls (HC) at Saarland University Medical School with the aim of investigating whether evidence of the previously described HIV-associated cerebral vasculopathy can also be found under modern antiretroviral therapy (ART) regimes with an easy-to-use method. Subjects of legal age were studied; history of CNS infection or stroke were exclusion criteria. The study was approved by the local ethics committee (Ethikkommission der Ärztekammer des Saarlandes, ethical vote No. 205/17) and conducted in accordance with the Declaration of Helsinki.

We prospectively examined 52 PLWH (45 males; infected with HIV for 11.9 years in the mean) and 28 HC controls (23 males) with median age of 52 and 51 years in 2019. In PLWH and HC group, the proportion of nicotine use (30.8% vs. 35.7%), arterial hypertension (17.3% vs 14.3%) and statin therapy (9.7% vs. 10.7%) were not significantly different. Mean CD4 lymphocyte count in PLWH was 649/μl (SD ± 271.12) under ART with good CSF penetration score ≥ 7 in 98.1%. 90.36% of PLWH had no detection of HIV copies in plasma. 80.3% received an ART with non-nucleoside reverse transcriptase inhibitor and integrase strand transfer inhibitor.

Color-coded duplex sonography (GE Logiq-E9) of the extracranial and intracranial arteries was performed, intima–media thickness (IMT) [3] in the common carotid artery was determined, and the pulsatility index (PI) [3] was calculated. Via transcranial ultrasound over the temporal acoustic window, we were able to rapidly detect the flow velocities in the MCA after 30 s of apnea without preceding deep inspiration. Cerebral reserve capacity (CRC) (%) was calculated from the quotient of the maximum flow velocity increase in apnea and mean flow velocity at rest (cm/s each) [4], Breath Holding-Index (BHI) as the quotient of flow increase and time of air holding [4, 5].

Additional (blinded) control MRIs revealed no silent cerebral infarcts and relevant differences in the frequency of cerebral microangiopathies (PLWH 27.5% vs HC 33.3%).

The flow velocities of the cerebral arteries (Table. 1a) were not different except for the posterior cerebral artery (but reduced number in the HC). There were no significant differences in median and mean of IMT, PI and CRC values between PLWH and HC (Table. 1b, c, d). There was no statistically significant correlation of IMT with CD-4 count, CD4 nadir, total cholesterol, LDL cholesterol, HDL cholesterol or triglycerides in PLWH.

**Table 1:** Ultrasound examinations, analyzed by Mann–Whitney *U* test

	PLWH, *n* = 52		HC, *n* = 28		
a. Flow velocity: artery (abbreviation), segment	*n*	mean (± sd) [cm/s]	*n*	mean (± sd) [cm/s]	*p*
Common carotid artery (CCA) right and left, extracranial	104	81.56 (± 17.44)	56	86.50 (± 20.98)	0.236
Internal carotid artery (ICA) right and left, extracranial	104	70.81 (± 11.79)	56	78.20 (± 48.84)	0.856
External carotid artery (ECA) right and left, extracranial	104	78.20 (± 21.20)	56	78.27 (± 16.02)	0.65
Vertebral artery (VA) right and left, V1, extracranial	102	65.51 (± 17.75)	54	64.43 (± 11.13)	0.801
Internal carotid artery (ICA) right and left, C1, intracranial	95	63.8 (± 14.15)	53	63.0 (± 16.18)	0.78
Anterior cerebral artery (ACA) right and left, A1, intracranial	89	70.6 (± 11.4)	47	71.1 (± 13.4)	0.755
Middle cerebral artery (MCA) right and left, M1, intracranial	99	90.1 (± 17.5)	55	93.0 (± 13.0	0.367
Posterior cerebral artery (PCA) right and left, P1, intracranial	45	52.4 (± 8.5)	21	63.5 (± 6.8)	** < 0.001**
Vertebral artery (VA) right and left, V4, intracranial	101	53.0 (± 12.0)	56	51.1 (± 9.4)	0.596
Basilar artery (BA), intracranial	52	59.1 (± 14.4)	28	61.0 (± 11.9)	0.276
b. Intima–media thickness (IMT)	*n*	mean (± sd) [mm]	*n*	mean (± sd) [mm]	*p*
IMT right, CCA	51	0.060 (± 0.015)	27	0.062 (± 0.021)	0.227
IMT left, CCA	51	0.065 (± 0.020)	27	0.061 (± 0.020)	0.704
c. Pulsatility index (PI)	*n*	mean (± sd)	*n*	mean (± sd)	*p*
PI right, ICA	43	0.95 (± 0.18)	21	1.03 (± 0.27)	0.751
PI left, ICA	39	1.01 (± 0.17)	22	1.03 (± 0.25)	0.212
d. Reserve capacity	*n*	mean (± sd)	*n*	mean (± sd)	*p*
Cerebral reserve capacity (CRC), MCA	50	32.82% (± 12.89)	28	34.46% (± 11.73)	0.579
Breath Holding-Index (BHI), MCA	50	1.094 (± 0.43)	28	1.149 (± 0.39)	

CRC did not correlate with age (Pearson’s *r* = 0.109; *p* = 0.581), but the values were lowered by 7.35% (*p* < 0.001) in PLWH and by 5.74% (*p* = 0.015) in HC compared to the known reference value [4] of 40.2%. There was no significant correlation between CRC and cholesterol fractions, triglycerides and current CD4 cell count. A comparison of the cohorts’ BHIs with newer reference values yielded no statistically significant difference [5]: PLWH (1.094 vs. 1.08; *p* = 0.818) and HC (1.149 vs. 1.26; *p* = 0.144).

## Conclusion

Ultrasound-detected stenosis, abnormal PI or IMT were not found, which differs from previous studies with lower CD4 counts [6]. Possibly, a high CD4 T-cell count seems to be associated with lower risk of atherosclerosis [7]. Cerebral reserve capacity, the lowering of which could indicate vasculopathy or increased risk of stroke, was also not significantly different [1]. We could not detect any evidence of cerebral vasculopathy in both groups. Although the prevalence of stroke remains elevated in PLWH, consistent therapy with modern ART, in addition to therapy for cerebrovascular risk factors, may reduce the inflammation leading to the development of HIV-associated vasculopathy. PLWH with normal CD4 cell counts do not appear to have an increased risk of stroke, although data for the risk of atherosclerosis with long-term treatment with modern ART such as integrase strand transfer inhibitors are still lacking. However, arterial ultrasound is also uncomplicatedly suitable for long-term clinical examinations.
